# High Prevalence of *Ancylostoma ceylanicum* Hookworm Infections in Humans, Cambodia, 2012

**DOI:** 10.3201/eid2006.131770

**Published:** 2014-06

**Authors:** Tawin Inpankaew, Fabian Schär, Anders Dalsgaard, Virak Khieu, Wissanuwat Chimnoi, Chamnan Chhoun, Daream Sok, Hanspeter Marti, Sinuon Muth, Peter Odermatt, Rebecca J. Traub

**Affiliations:** University of Copenhagen, Copenhagen, Denmark (T. Inpankaew, A. Dalsgaard); Kasetsart University, Bangkok, Thailand (T. Inpankaew, W. Chimnoi);; Swiss Tropical and Public Health Institute, Basel, Switzerland (F. Schär, V. Khieu, H. Marti, P. Odermatt);; University of Basel, Basel (F. Schär, V. Khieu, H. Marti, P. Odermatt);; National Center for Parasitology, Entomology and Malaria Control, Phnom Penh, Cambodia (V. Khieu, S. Muth);; Fisheries Administration, Phnom Penh (C. Chhoun, D. Sok);; The University of Queensland, Gatton, Queensland, Australia (R.J. Traub);; University of Melbourne, Parkville, Victoria, Australia (R.J. Traub)

**Keywords:** hookworm, Ancylostoma ceylanicum, zoonosis, Cambodia, humans

## Abstract

*Ancylostoma ceylanicum*, a hookworm of canids and felids in Asia, is becoming the second most common hookworm infecting humans. In 2012, we investigated the prevalence and infection dynamics of and risk factors for hookworm infections in humans and dogs in a rural Cambodian village. Over 57% of the population was infected with hookworms; of those, 52% harbored *A. ceylanicum* hookworms. The greatest intensities of *A. ceylanicum* eggs were in persons 21-30 years of age. Over 90% of dogs also harbored *A. ceylanicum* hookworms. Characterization of the cytochrome oxidase-1 gene divided isolates of *A. ceylanicum* hookworms into 2 groups, 1 containing isolates from humans only and the other a mix of isolates from humans and animals. We hypothesize that preventative chemotherapy in the absence of concurrent hygiene and animal health programs may be a factor leading to emergence of *A. ceylanicum* infections; thus, we advocate for a One Health approach to control this zoonosis.

## Abstract 2

Preventative chemotherapy without community hygiene and animal health programs may be leading to emergence of this zoonosis.

Human infections with *Necator americanus* and *Ancylostoma duodenale* hookworms continue to be recognized as a leading cause of iron deficiency anemia and protein malnutrition in developing countries (*1*). On the basis of parasitologic surveys of fecal samples, hookworms are estimated to infect 576–740 million persons globally, and over half of the infections occur in Asia and the Pacific regions (*2*). Recent molecular-based epidemiologic surveys have shown *Ancylostoma ceylanicum* to be the second most common hookworm species infecting humans in Asia. In Thailand, Laos, and Malaysia, 6%–23% of persons positive for hookworm eggs were infected with *A. ceylanicum* helminths (*3*–*6*). There are an estimated 19–73 million *A. ceylanicum* hookworm–infected persons in regions where this zoonotic helminth is known to be endemic (*7*). Dogs and cats act as natural reservoirs for hookworm transmission to humans, and the prevalence of *A. ceylanicum* hookworms in these animals ranges from 24% to 92% in the Asia-Pacific region (*6*,*8*–*10*). Much like anthroponotic helminths, *A. ceylanicum* hookworms have the potential to produce clinical symptoms of ground itch (a pruritic papular hypersensitivity response caused by the entry of helminths into the skin), epigastric pain, diarrhea, and anemia in humans (*11*–*15*). However, despite these reports, relatively little is known about the clinical significance and infection dynamics of this zoonotic hookworm in humans, dogs, and cats. Differentiation of the genus of hookworms infecting humans is imperative because each species varies in its biology, life cycle, pathophysiology, and epidemiology, and these differences have key implications when assessing hookworm-associated illnesses and establishing control measures.

The internal transcribed spacer (ITS) –1 and –2 regions and the 5.8S region have been used to detect and characterize hookworm infections directly from eggs in human and animal feces (*6*,*10*,*16*,*17*). In addition, sequencing of the cytochrome c oxidase subunit 1 (*cox1*) gene has been successfully used to establish intraspecies genetic differences of many strongylid nematodes, including hookworms (*18*–*21*).

The aim of our study was to determine the prevalence, associated risk factors, and infection dynamics of hookworm species infection in humans and dogs living in a rural Cambodian village. To carry out this investigation, we used a combination of conventional parasitologic and molecular epidemiologic approaches.

## Materials and Methods

### Study Site and Sample Collection

The study was conducted in May 2012 in Dong, a rural village in Rovieng District, Preah Vihear Province, Cambodia. Preah Vihear Province is located in northern Cambodia, bordering Thailand and Laos (13°47′N 104°58′E). The climate is tropical; temperatures are warm and hot all year round, and seasons alternate between dry and wet. Subsistence farming (rice, vegetables, and fish) constitutes the primary source of income for the community. Drinking water is sourced from wells, well pumps, and rain water tanks, and just over half of the households own a latrine. All household electricity is battery or generator powered. Approximately half the households feed semidomesticated, free-roaming community dogs. These dogs are allowed to defecate indiscriminately within the village or outside the homes of their owners.

The study protocol was approved by the Ethics Committee of the Canton of Basel and Baselland, Switzerland, and the National Ethics Committee Health Research, Ministry of Health, Cambodia. Dong, the village selected for study, had previously been categorized as having endemic soil-transmitted helminths (*22*). According to the treatment guidelines of the Cambodian helminths control program, all children attending primary school in the village were administered albendazole (400 mg) and mebendazole (500 mg) twice a year. At completion of the study, all participants who were found positive for *Strongyloides* spp. were treated with ivermectin (200 μg/kg body weight), and participants infected with other soil-transmitted helminths were treated with albendazole (400 mg).

A cross-section of 67 households was randomly selected from a list provided by the Dong village authority. A total of 218 persons from those households were enrolled in the study. Of the 218 persons, 99 (45.4%) were male. The average age of participants was 30.0 years (range 2–84); female participants were marginally older, on average, than male participants (30.3 vs. 29.8 years of age). On the first day of the study, informed consent was obtained from the enrolled participants, and questionnaires were administered during interviews. Interviews with children (i.e., participants 2–17 years of age) were conducted with the assistance of a parent or legal guardian. All study participants responded to a questionnaire covering demographics, dietary habits, personal hygiene, and level of household income and assets.

Prelabeled stool containers were distributed to the 218 study participants for collection of feces on the second morning of the study. Fecal samples were collected from participants’ dogs (N = 94), when applicable. Samples (≈3–5 g) from dogs were collected directly from the rectum at time of the participant interview and placed into a sterile plastic container. If insufficient stool was obtained from a dog, the animal was confined within the owner’s property and resampled on the second morning of the study. All fecal samples were chilled immediately in a cool box and transported to a laboratory in Rovieng Health Center (Rovieng District, Preah Vihear Province) within 2 h after collection. After fecal samples arrived at the laboratory, a minimum of 2 g of each sample was placed into a 15-mL centrifuge tube containing 10% formaldehyde for parasitologic analysis, and 1–2 g of each sample was placed into a 15-mL centrifuge tube containing 2.5% potassium dichromate for molecular analysis. These samples were then shipped at room temperature to the School of Veterinary Science, University of Queensland, Gatton, Queensland, Australia, for further analysis.

### Parasitologic Procedures

All fecal samples were examined by microscope. The relative intensity of hookworm infection, in eggs per gram, was determined by floatation, using a sodium nitrate solution (specific gravity 1.20) (*23*).

### DNA Extraction

Genomic DNA was extracted directly from fecal samples by using the PowerSoil DNA Isolation Kit (Mo Bio, Carlsbad, CA, USA) according to the manufacturer’s instructions, with the exception that fecal samples were subjected to a 5-min disruption by using 0.5-mm Zirconia/Silica beads (BioSpec Products, Inc., Bartlesville, OK, USA) instead of the beads provided by the manufacturer. Final elution of DNA was made in 100 μL of elution buffer. The extracted DNA was stored at −20°C until required for PCR amplification.

### Molecular Characterization of Hookworm species in Humans

PCR was conducted by using primers RTHW1F and RTHW1R (*10*) in 25-μL volumes; each final reaction contained 1× CoralLoad PCR Buffer (QIAGEN Pty Ltd, Hilden, Germany), 12.5 pmol of each primer, 0.5 U of HotStar Taq DNA Polymerase (QIAGEN), and 2 μL of DNA. The cycling conditions were the same as the published protocol (*10*) except for an initial denaturation of 5 min at 95°C. A positive control of *N. americanus* and *A. ceylanicum* hookworms and negative controls of distilled water were included in each run. PCR amplicons that were ≈380 bp in size, corresponding to *Ancylostoma* spp. hookworms, were purified by using the PureLink Quick PCR Purification Kit (Life Technologies, Carlsbad, CA, USA) and submitted to the University of Queensland Animal Genetics Laboratory, Gatton, for bidirectional DNA sequencing.

### Molecular Characterization of Hookworms species in Dogs

PCR–restriction fragment length polymorphism (RFLP) characterization of hookworms from dogs was carried out as described (*17*,*24*). In brief, RTGHFI and RTABCR1 primers were used to amplify a 545-bp region of ITS-1, 5.8S, and ITS-2 of *A*. *caninum*, *A. ceylanicum*, and *Uncinaria stenocephala* hookworms. In a separate PCR, a 673-bp region of an *A*. *braziliense* hookworm was amplified by using RTGHF1 and a specific reverse primer, RTAYR1. Both PCR reactions consisted of 1× CoralLoad PCR Buffer (QIAGEN), 12.5 pmol of each primer, 0.2 μL of 20 mg/mL bovine serum albumin, 2 μL DNA, and 1 U of HotStar Taq Polymerase (QIAGEN) in a 25-μL reaction. The cycling conditions were as published (*17*,*24*), except for an initial denaturation time of 5 min at 95°C. Amplified PCR product (10 μL; RTGHF1/RTABCR1) was digested with *HinF*I and *Rsa*I endonucleases in separate reactions at 37°C for 3 h. The RFLP patterns generated by each sample were then compared to the expected RFLP profiles for each hookworm species.

### PCR and DNA Sequencing of *cox1* of *A. ceylanicum* Hookworm

Samples from dogs and humans that were positive for *A. ceylanicum* hookworms were further characterized to a haplotype level by analysis of the mitochondrial gene (*cox1*). AceyCOX1F (5′-GCTTTTGGTATTGTA-AGACAG-3′) and AceyCOX1R (5′- CTAACAACATAATAAG-TATCATG-3′) were specifically designed to amplify a 377-bp region of the *cox1* gene of *A. ceylanicum* hookworm. The PCR was carried out in 25-μL volumes, with each reaction containing 1× CoralLoad PCR Buffer, 12.5 pmol of each primer, 0.5 U of HotStar Taq DNA polymerase, and 2 μL of DNA. The cycling conditions were 95°C for 5 min, followed by 50 cycles at 94°C for 30 s, 58°C for 30 s, 72°C for 30 s, and a final extension at 72°C for 7 min. A positive control of *A. ceylanicum* hookworm and a negative control of distilled water were included in the run. PCR-positive samples were purified by using the PureLink Quick PCR Purification Kit according to the manufacturer’s protocol. Bidirectional DNA sequencing was performed by the University of Queensland Animal Genetics Laboratory.

### Phylogenetic Analyses

DNA sequences were analyzed by using the Finch TV version 1.4.0 trace viewer (Geospiza, Inc., Seattle, WA, USA) and aligned by using BioEdit version 7.2.0 (www.mbio.ncsu.edu/BioEdit/bioedit.html) together with the *cox1* gene sequences from the following hookworm species: *A. ceylanicum* Malaysia isolates (GenBank accession nos. KC247727– KC247745, Pos Iskandar [Human] and Sg Bumbun [Human]); *A. caninum* and *A. duodenale* (GenBank accession nos. NC012309 and NC003415, respectively); and *A. ceylanicum* Thailand genotype (GenBank accession no. KF896595). Neighbor-joining analyses were conducted by using Tamura-Nei parameter distance estimates, and the tree was constructed by using Mega4.1 (www.megasoftware.net). Bootstrap analyses were conducted using 1,000 replicates.

### Statistical Analyses

We used STATA version 12 (StataCorp LP, College Station, TX, USA) for data entry and statistical analyses. The prevalence of hookworm infection was calculated by using descriptive statistics for microscopy and molecular results. A univariate model was used to assess potential risk factors associated with hookworm infection; odds ratios and 95% CI were reported. The level of statistical significance was set at p<0.05. Factors that were significant in univariate analysis were evaluated by multivariate analysis, when applicable.

## Results

### Prevalence of Hookworm Infection

The prevalence of hookworm infection among the 218 persons tested in Dong village was 26.6% (58/218) as determined by microscopic examination and 57.4% (124/218) as determined by PCR based on amplification of the partial ITS gene. Among dogs, 80.9% (76/94) were positive for hookworms by microscopic examination, and 95.7% (90/94) were positive by PCR based on amplification of the partial ITS gene.

### Molecular Characterization of Hookworm Species

Of the 124 persons with positive samples, 64 (51.6%) harbored *A. ceylanicum* hookworms; 57 (89.0%) of these infections were single infections. An equal percentage of persons, 64 (51.6%), were infected with *N. americanus* hookworms, mostly as single infections (59/64 [92.2%]), and 4 (3.2%) persons were infected with *A. duodenale* hookworms (Table).

**Table:** Hookworm species found in humans and dogs, Dong village, Rovieng District, Preah Vihear Province, Cambodia, 2012*

Infected host, hookworm species	No. (%) positive
Humans	
* Necator americanus*	59 (47.6)
* Ancylostoma ceylanicum*	57 (46.0)
* A. duodenale*	1 (0.8)
*N. americanus* and *A. ceylanicum*	4 (3.2)
*A. ceylanicum* and *A. duodenale*	2 (1.6)
*N. americanus* and *A. ceylanicum* and *A. duodenale*	1 (0.8)
*N. americanus* and *A. duodenale*	0
Total	124 (100.)
Dogs	
* A. ceylanicum*	81 (90.0)
* A. caninum*	5 (5.6)
*A. ceylanicum* and *A. caninum*	3 (3.3)
*A. ceylanicum* and *N. americanus*	1 (1.1)
Total	90 (100.0)

*The presence of hookworms was determined by PCR amplification of the internal transcribed spacer–1 and –2 regions and the 5.8S region and by DNA sequencing.

Of the 90 dogs with positive samples, 85 (94.4%) were infected with *A. ceylanicum* hookworms, mostly (81/85 [95.3%]) as single infections, and 8 (8.9%) were infected with *A. caninum* hookworms. One dog was found to be shedding *N. americanus* eggs (Table).

### Phylogenetic Analysis of *cox1* Gene of *A. ceylanicum*

Of 68 human and 82 dog samples positive for hookworms, 28 (41.2%) and 65 (79.3%), respectively, were successfully amplified at the *cox1* gene. Of these, 21 PCR-positive amplicons from human samples and 27 PCR-positive amplicons from dog samples were randomly selected for DNA sequencing and subsequent phylogenetic analysis.

The phylogenetic tree distinctly separated into 3 clusters; the *A. ceylanicum* hookworm isolates grouped together and were genetically distinct from *A. caninum* hookworm isolates (GenBank accession nos. NC012309 and FJ483518) and *A. duodenale* hookworm isolates (GenBank accession nos. NC003415 and AJ417718). Within *A. ceylanicum* hookworm isolates, there was strong bootstrap support (100%) for the division of isolates from various geographic locations into 2 clades. The first clade comprised 4 human isolates, 1 from the current study in Cambodia (Human 19) and 3 previously reported human isolates from Malaysia (GenBank accession no. KC772445; Human [Sg Bumbun]; Human [Gurney] and Human [Pos Iskandar]). The second clade comprised a mix of isolates from humans (n = 20) and dogs (n = 27) from villages in Cambodia; humans (n = 5), dogs (n = 11), and cats (n = 2) from Malaysia (*21*); and 1 dog in Thailand (GenBank accession no. KF896595). For human- and dog-derived *A. ceylanicum* hookworms, representative DNA sequences at each *cox1* haplotype were submitted to GenBank under accession nos. KF896596–KF896605 (see sequences marked with asterisks in the Technical Appendix Figure).

### Age-related Prevalence and Intensity of *N. americanus* and *A. ceylanicum* Hookworm Infections

The prevalence of *N. americanus* hookworms peaked in persons 31–50 years of age, whereas the prevalence of *A. ceylanicum* hookworms peaked in persons 15–20 years age and again in persons 31–50 years of age (Figure). The highest egg intensities for single infections attributed to *N. americanus* and *A. ceylanicum* hookworms occurred in persons 21–30 years of age (Figure).

**Figure:**
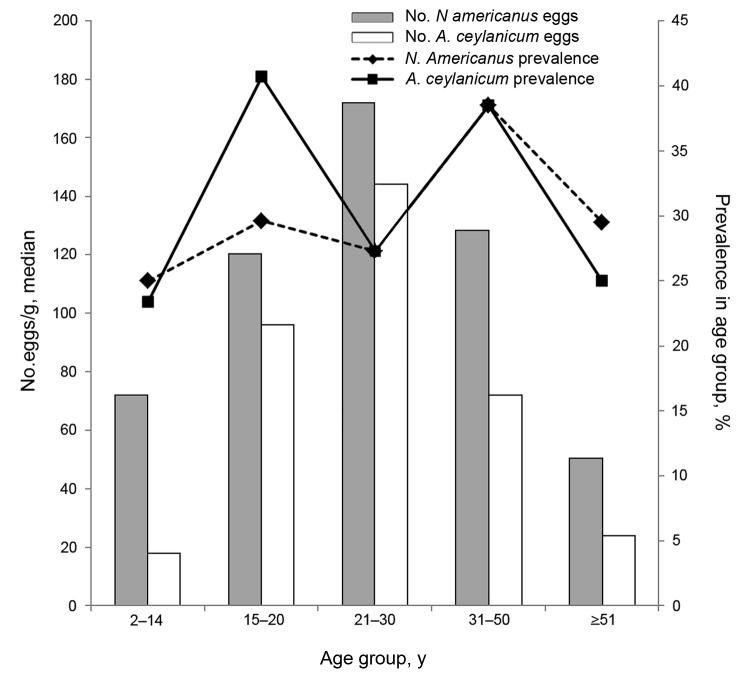
Prevalence and intensity (eggs per gram) of *Necator americanus* and *Ancylostoma ceylanicum* hookworm infections in humans of different ages in rural Dong village, Rovieng District, Preah Vihear Province, Cambodia, 2012.

### Risk Factors Associated with Hookworm Infection of Humans and Dogs

The results of regression analysis showed an increased risk for hookworm infection in persons who did not wear shoes while defecating (odds ratio 6.0, 95% CI 1.1–28.6; p = 0.038). No significant associations were found between the prevalence and intensity of hookworms by age group, sex, household income, or dietary practices. No risk factors of significance were associated with hookworm infection in dogs.

## Discussion

In this study, zoonotic ancylostomiasis caused by *A. ceylanicum* hookworms was found to be highly endemic among humans in Dong village, Preah Vihear Province, Cambodia; community dogs were the likely zoonotic reservoir. This finding is in stark contrast to the consistent finding by other molecular-based prevalence studies in the region that *N. americanus* is the predominant hookworm species in humans, followed by *A. ceylanicum* and *A. duodenale* hookworms (*3*,*5*,*6*,*10*). PCR proved a superior alternative to microscopy-based techniques for the detection of hookworms in fecal samples (*25*,*26*). In Dong village, the prevalence of *A. ceylanicum* hookworms matched that of their anthroponotic counterpart, *N. americanus* hookworms, and infections with *A. ceylanicum* hookworms substantially out-numbered those with *A. duodenale* hookworms. In addition, most infected persons harbored single-species hookworm infections; just over 10% of hookworm-positive persons had mixed-species infections. These results raise questions about the potential infection dynamics between hookworm species within individual hosts. Our study supports an earlier hypothesis (*7*) that anthroponotic hookworms may have a cross-protective role in expelling and preventing the subsequent establishment of *A. ceylanicum* hookworms via a T helper 2 cell response (*27*). The major immunologic action against incoming L3 larvae (infective filariform larvae) and L4 larvae (final larval stage within the intestine) is regulated by the infection itself (*28*). Thus, the presence of a stable and long-lived (3–6 years) infection with anthroponotic species (*29*) may play a role in providing an unsuitable environment for the establishment of incoming larvae of another closely related (albeit potentially shorter-lived and suboptimally host adapted) species—in this case, *A. ceylanicum* hookworms. Reduced burdens of anthroponotic hookworm species may also have the added advantage of easing density-dependent intraspecific competition for limited resources within the intestinal niche (*30*), leading to an opportunistic establishment of *A. ceylanicum* hookworms. Although data on the natural life span of *A. ceylanicum* hookworms in humans do not exist, infections in Dutch servicemen 5 months after their return from New Guinea (*31*) suggest that chronic infections with this hookworm may occur.

The initiating or causal factor for the emergence of highly endemic levels of monospecific infections with *A. ceylanicum* hookworms in Dong village remains unclear. In this study, potential causal factors for human infection are likely related to the high levels of *A. ceylanicum* hookworm infections in community dogs. In rural Malaysia, close contact with community dogs and cats was shown often to be associated with human infection with *A. ceylanicum* hookworms (*6*). In Dong village, dogs were reported to defecate indiscriminately in environments shared with humans, leading to widespread environmental contamination with infective *A. ceylanicum* hookworm larvae. For humans, defecating while bare foot was shown to be the most significant risk factor for infection with both species of hookworms. Whether these factors, coupled with the administration of preventative chemotherapy, led to an increased opportunity for the *A. ceylanicum* hookworm to replace the niche of its anthroponotic competitors remains unanswered. Either way, integrated control programs aimed at combining chemotherapeutic interventions with improvements in community hygiene and animal health programs will aid in curbing this potentially opportunistic zoonosis.

Molecular epidemiologic data gathered from characterization of the *cox1* gene of *A. ceylanicum* hookworms strongly support previous findings (*21*) that *A. ceylanicum* hookworm isolates from humans and animals formed 2 genetically distinct groups, 1 comprising isolates specific to humans and the other comprising isolates from humans, dogs, and cats. Most *A. ceylanicum* hookworm isolates from humans in Dong village clustered within the zoonotic haplotype, confirming that transmission from dogs to humans has occurred. Genetic groups inferred by the *cox1* gene of *A. ceylanicum* hookworms were found to be independent of geographic source. Whether the 2 primary haplotypes differ in biologic, epidemiologic, and pathophysiologic characteristic warrants further investigation.

The transmission dynamics of *A. ceylanicum* hookworms in humans of different ages largely paralleled that of *N. americanus* hookworms: persons 21–30 years of age excreted the highest number of eggs. This highly unexpected finding has key implications. First, this finding suggests that the previous classification of *A. ceylanicum* as an abnormal and minor hookworm of humans (*32*) no longer stands. Second, monospecific infections of humans with <100 *A. ceylanicum* worms have been reported to cause anemia, even in well-nourished persons (*14*,*33*). Thus, attention must be directed to *A. ceylanicum* infection as a major cause of human illness in areas where this zoonosis is endemic.

The zoonotic helminth *A. ceylanicum* can no longer be classified as an abnormal hookworm of humans. Although previous studies have reported this hookworm’s emergence as the second most common human hookworm species in Southeast Asia, our study demonstrates its ability to infect humans at prevalence and intensity levels at par with that of its anthroponotic competitor, the *N. americanus* hookworm. We hypothesize that expansion of preventative chemotherapy in the absence of concurrent hygiene and animal health programs is a potential causal factor for the emergence of this zoonosis. Attention must be directed to the effects of *A. ceylanicum* hookworm infections on human health, and a One Health approach should be adopted for the control of this zoonosis.

Technical Appendix Phylogenetic tree of *Ancylostoma ceylanicum* hookworms from 21 humans and 27 dogs in Cambodia together with reference isolates from Malaysia and Thailand.
