# Supplementary material for: High Prevalence of Ancylostoma ceylanicum Hookworm Infections in Humans, Cambodia, 2012
Source: Emerg Infect Dis. 2014 Jun;20(6):976–82. doi: 10.3201/eid2006.131770 (PMC4036766; doi:10.3201/eid2006.131770)
Supplement: Technical Appendix — Phylogenetic tree of Ancylostoma ceylanicum hookworms from 21 humans and 27 dogs in Cambodia together with reference isolates from Malaysia and Thailand. [file 13-1770-Techapp-s1.pdf]

Article DOI: http://dx.doi.org/10.3201/eid2006.131770

High Prevalence of *Ancylostoma ceylanicum* Hookworm Infections in Humans, Cambodia, 2012

Technical Appendix

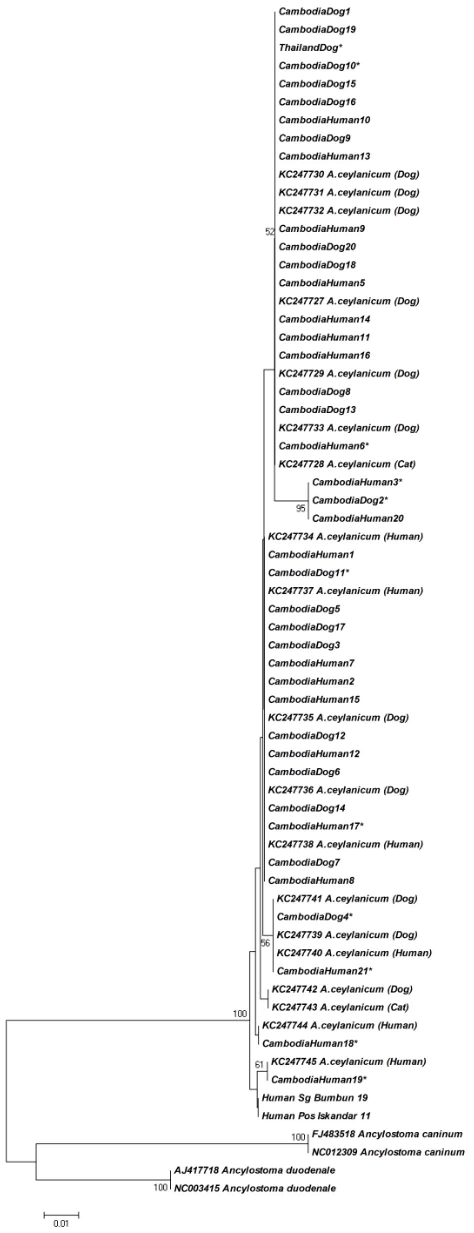

Technical Appendix Figure. Phylogenetic tree obtained from neighbor-joining analysis of cox-1 gene amplicons (377 bp) of *Ancylostoma ceylanicum* hookworms sourced from 21 humans and 27 dogs in Cambodia together with reference isolates from Malaysia and Thailand sourced from GenBank (KC247727–45).
